# Astrocyte morphology: Diversity, plasticity, and role in neurological diseases

**DOI:** 10.1111/cns.13123

**Published:** 2019-03-30

**Authors:** Bin Zhou, Yun‐Xia Zuo, Ruo‐Tian Jiang

**Affiliations:** ^1^ Laboratory of Anesthesia and Critical Care Medicine, Department of Anesthesiology, Translational Neuroscience Center West China Hospital, Sichuan University Chengdu China

**Keywords:** astrocyte, diversity, morphology, plasticity, tripartite synapse

## Abstract

Astrocytes are the most abundant glial cells in the central nervous system (CNS) and participate in synaptic, circuit, and behavioral functions. The well‐developed protoplasmic astrocytes contain numerous processes forming well‐delineated bushy territories that overlap by as little as 5% at their boundaries. This highly complex morphology, with up to approximately 80% of the cell's membrane constituted by fine processes with dimensions on the tens of nanometer scale and high surface area to volume ratios, comes in contact with synapses, blood vessels, and other glial cells. Recent progress is challenging the conventional view that astrocytes are morphologically homogeneous throughout the brain; instead, they display circuit‐ and region‐specific morphological diversity that may contribute to the heterogeneous astrocyte‐neuron spatiotemporal interplay in different brain areas. Further, the fine structure of astrocytes is found to be highly plastic and activity‐dependent. We are beginning to understand how astrocyte structural plasticity contributes to brain functions. The change/loss of astrocyte morphology, traditionally known as a hallmark for reactive astrogliosis, is a common pathological feature in many neurological disorders. However, recent data suggest the fine structural deficits preceding reactive astrogliosis may drive disease progression. This review summarizes recent advances in astrocyte morphological diversity, plasticity, and disease‐related deficits.

## 1 INTRODUCTION

In mammalian brains, astrocytes represent the most abundant cell type accounting for approximately 20% ~ 40% of the total number of brain cells.^1^ Protoplasmic and fibrous astrocytes are two major astroglial types that are prominently found in gray matter and white matter of both the cerebrum and spinal cord, respectively, and are distinct in morphology.^2^, ^3^, ^4^, ^5^ In addition, there are some specialized astroglial cells such as Müller cells in the retina and Bergmann glial cells in the cerebellum.^6^ The protoplasmic astrocytes are in close contact with pre‐ and postsynaptic compartments and are actively engaged in synaptic development and function in most parts of mammalian brain.^7^ The focus of this review mainly deals with protoplasmic astrocytes in the context of synaptic physiology. Individual protoplasmic astrocytes have very complex spongiform shapes with diameters of ~40‐60 μm and volumes on the order of 10^4^ μm.^8^, ^9^, ^10^ Astrocyte processes can be further classified using the following terminology based on their sizes and locations: branches, branchlets, leaflets, and end feet.^11^ Branches and branchlets are the stem processes and secondary (and tertiary processes), respectively. Leaflets, also termed as peripheral fine processes or perisynaptic astrocytes processes (PAPs), are the finest processes which cannot be reliably imaged by light microscopy; End feet are specialized and polarized astrocyte structures in contact with blood vessels. Compared to rodent astrocytes, human protoplasmic astrocytes are found to be ~2.5 times larger in diameter, ~16.5 times larger in volume,^12^ and 10 times increase in number of GFAP‐positive processes which are radially and symmetrically in all directions from soma.^13^ Importantly, individual human astrocyte covers ~2 million synapses,^13^ with a synaptic density of ~1100 million synapses mm^‐3^. In addition, there is a type of glia, with several (up to 5) very long (up to 1 mm) unbranched processes and called varicose projection astrocytes, exists only in human brain.^12^ The increased astrocyte complexity in human brain account, at least in part, for human intelligence.^13^, ^14^ Recent studies using morphological reconstruction along with transcriptomic and proteomic analysis suggest astrocytes exhibit regional and microenvironmental divesity,^10^, ^15^ and astrocyte PAPs may exhibit local synaptic activity‐, brain state‐, or behavior‐dependent structural remodeling.^16^, ^17^, ^18^, ^19^ We are only beginning to understand the molecular basis of the astrocyte structural diversity and plasticity, yet much remains unknown regarding how it contributes to synapse, neural circuit, and behavior functions. Reactive astrogliosis is detected in nearly all kinds of injuries and neurological disorders, usually in the late stage of the neurodegeneration.^20^, ^21^, ^22^ However, emerging evidence suggests that astrocytes display morphological deficits preceding astrogliosis that may contribute to the progression of neurological diseases.^23^

## 2 MORPHOLOGICAL DIVERSITY OF ASTROCYTES

### 2.1 Phenotypes

Recent progress has shown that protoplasmic astrocytes in rodent CNS appear to be regional and layer‐specific diverse.^10^, ^15^ Within a single astrocyte territory, the number of neuronal cell bodies and synapse density vary dramatically: ~50 700 excitatory synapses and about 20 neuronal cell bodies in the striatum, ~95 200 excitatory synapses and at most 1 neuronal cell body in the hippocampus,^10^ and 300‐600 neuronal dendrites and 4‐8 neuronal cell bodies in the cortex.^24^ Within the hippocampal CA1 s.r (stratum radiatum) of rats, protoplasmic astrocytes also display morphological diversity, that is, fusiform, elongated, and spherical cells were distinguished.^8^ Similarly, in the hippocampal dentate gyrus (DG) of albino Swiss mice, two morphological phenotypes, that is, type I and type II, astrocytes were found. Type I astrocytes exhibited significantly higher values of morphological complexity which were more sensitive to aging and environmental influences as compared with type II.^25^ In mouse somatosensory cortex, astrocytes in different layers exhibited distinct morphologies. Lanjakornsiripan et al^15^ reported that astrocytes in layer II/III tended to elongate radially, in contrast, layer VI astrocytes tended to elongate tangentially. Significant differences in territory volume were also detected among different layers and brain regions.^10^ Whether or not these phenotypes produce functional heterogeneity is unknown.

Only a proportion of synapses are enwrapped by astrocytes. In the mouse somatosensory cortex, the ensheathment of synaptic clefts by astrocytes in layer II/III (about 80%) is greater than those in layer VI (about 40%).^15^ In the layer IV, 90% of spines are reported to be contacted by astrocytes, and most of them (about 68%) have astrocytes contacting the synaptic clefts.^26^ Cross‐regional diversity is also reported. In the rat neocortex, only 29%‐56% of excitatory synapses are ensheathed by astrocyte processes.^16^, ^26^ In contrast, in hippocampal CA1 s.r, PAPs are present in approximately 57 to 62% of synapses,^27^, ^28^ and synapses are larger when astrocyte processes are present at the axon‐spine interface (ASI).^27^ Given that synapses are larger when perisynaptic astrocyte is present than when it is absent,^27^ it is possible that these synapses are functionally different from those without astrocyte ensheathment.

The perimeter of ASI surrounded by PAPs is another parameter to reflect the spatial interactions of astrocytes and synapses. In the hippocampal CA1 s.r of rats, strong correlation exists between the astrocyte‐free length of the ASI perimeter and postsynaptic density (PSD) area. Synapses without PAPs at the ASI have longer unopposed lengths than those with PAPs at the ASI. This suggests that larger synapses, with greater capacity for neurotransmitter release, have a longer interface from which glutamate and other substances can escape from or enter into the synapse.^27^ On the contrary, Gavrilov et al^29^ reported that the astrocytic coverage of small and larger spines is similar in hippocampal neuropil. In contrast to hippocampal CA1 s.r, PAPs in CA3 virtually isolate synapses from the surrounding tissue, thus, make spillover almost impossible.^30^ In the cerebellum, the perimeter of synaptic clefts ensheathed by astrocytes in synapses formed by climbing fibers (CF) which is approximate 87% on average, is much higher than those formed by parallel fibers (PF) (about 65% on average).^31^ Astrocytic coverage of synapses represents a physical barrier to molecules diffusing into the extracellular space (ECS), which therefore is essential to limit the spillover of synaptic glutamate and other neuroactive substances.^32^, ^33^ Thus, diversity of astrocytic coverage represents the differences in the tendency of extrasynaptic substances spillover, which consequently regulates the heterosynaptic transmission and intercellular communication among different brain areas or layers. Moreover, astrocyte‐synapse interactions also affect the astrocytic glutamate release and uptake, which shape the pathway‐specific alterations in heterogeneous synaptic transmission.^34^

The distances between astrocyte membranes and synaptic clefts, calculated by distances between PAPs and PSD, vary from direct contact to hundreds of nanometers,^35^, ^36^ correlated with spine types. Astrocytes selectively approach synapses on thin dendritic spines, for example, astrocyte protrusions occur twice as close to the PSDs on thin dendritic spines when compared to those on mushroom spines in the hippocampal DG of mice.^36^ In addition, regional diversity also exists. The striatal astrocyte processes are further away from PSD centers for all types of spines in comparison with those in hippocampal CA1. The number of astrocyte‐contacted synaptic interfaces in hippocampal CA1 s.r is larger than those in striatum.^10^ The spatial proximity of PAPs to synaptic clefts controls the functional efficiency of astrocyte transporters, such as glial glutamate transporters (GLTs: GLT1 and GLAST), hence modulates synaptic transmission.^37^ For example, PAPs that deeply invade into synaptic clefts (> 150 nm) show higher GLTs efficiency than those without invading, which decreases the excitatory synaptic transmission.^37^ In summary, the way how astrocyte PAPs present, approach, or contact with synapses may affect synaptic strength, plasticity, and efficiency at a single synapse, and it varies among single synapses within the same neural microcircuit and among different brain regions.

### 2.2 Molecular basis

How their morphological diversity is established remains incompletely understood. Layer‐specific differences in astrocyte properties are abolished in *reeler *and *Dab1* conditional knockout mice, where the proper neuronal layers are diminished,^15^ suggesting neuronal layers guide astrocyte morphogenesis. Another study that highlights the neuronal cues for astrocyte morphology/morphogenesis is that Stogsdill et al reported that direct contact with neuronal processes, through the interaction of astrocytic neuroligin (NL) family proteins, that is, NL1, NL2, and NL3, with neuronal neurexins is required for proper astrocyte morphology/morphogenesis.^38^ Interestingly, the three β‐isoforms of neurexins, binding to all three NLs,^39^ show regional‐specific distribution pattern.^40^ In all, it indicates that the regional‐specific expression of astrocyte‐neuron signaling molecules such as neuroligin and neurexins plays a role in astrocyte morphological heterogeneity.

The gap junction protein connexin (Cx) 30 is another emerging candidate for the regulation of astrocyte morphology. In rodent brain, connexin 30 is selectively expressed in gray matter astrocytes and colocalizes with connexin 43 at gap junctions.^41^ Connexin 30 regulates cell adhesion and migration, which occurs independently of its channel function. In vitro experiments reveal that connexin 30 sets the orientation of astroglial motile protrusions via modulation of the laminin/β1 integrin/ Cdc42 polarity pathway. In vivo, connexin 30 also contributes to the establishment of hippocampal astrocyte polarity during postnatal brain maturation.^42^ Moreover, connexin 30 controls the insertion of astroglial processes into synaptic clefts, hence modulates glutamate uptake.^37^ Both connexins are found to be enriched within barrels, compared with septa and other cortical layers.^43^ This regional diversity of connexin expression may contribute to morphological heterogeneity of astrocytes.

In *Drosophila*, astrocytes show similarity to the mammalian protoplasmic astrocytes.^44^, ^45^ The astrocyte outgrowth into synaptic regions and the size of individual astrocytes are mediated by the Heartless (Htl) fibroblast growth factor (FGF) receptor signaling pathway. Pyramus and Thisbe, the only known FGFs activating Htl and produced by neurons, direct astrocyte processes to ramify specifically in CNS synaptic regions.^45^ The FGF signaling pathway is also required for extension of ensheathing glia process to insulate each neuropil compartment in the antennal lobe^46^ and for branch extension of astrocyte‐like medulla neuropil glia (MNG) into the synaptic neuropil in the visual system of *Drosophila.* 47 Apart from the FGFs pathway, the focal adhesion (FA) molecules, such as Tensin, β‐integrin, Talin, focal adhesion kinase (FAK), or matrix metalloproteinase 1 (Mmp1), are also critical regulators of astrocyte morphological development.^48^ Thus, the FGF signaling together with FAs are suggestive cues for morphogenesis and morphological heterogeneity of *Drosophila* glia.

**Figure 1:**
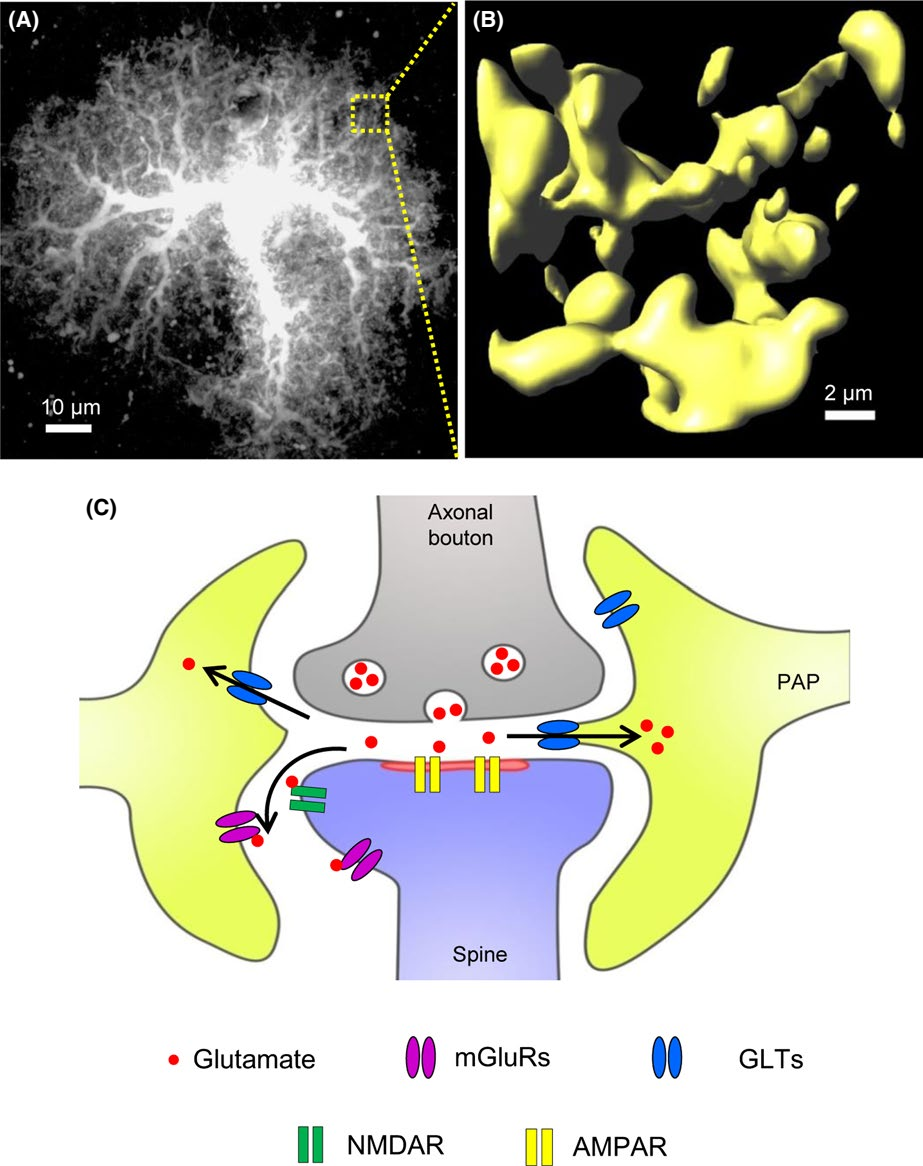
Protoplasmic astrocyte has very complex morphology with PAPs ensheathing the synapse. A, Representative confocal image of a protoplasmic astrocyte in the somatosensory cortex of adult mouse. B, Representative 3D reconstruction of astrocytic peripheral fine processes within a given ROI (5 μm × 5 μm × 5 μm). C, Cartoon of tripartite synapse, where PAPs approach or invade the synaptic cleft. The depth of astrocyte invasion controls the functional efficacy of GLTs which consequently affects the synaptic transmission. AMPAR, α‐amino‐3‐hydroxy‐5‐methyl‐4‐isoxazolepropionic acid receptor; GLTs, glial glutamate transporters, including GLT1 and GLAST; mGluRs, metabotropic glutamate receptors; NMDAR, N‐Methyl‐D‐aspartic acid receptor; PSD, postsynaptic density. Images in A and B are provided by authors

Besides aforementioned molecular proofs, at the light microscope level, astrocytes show the diversity from each other in shape, size, and orientation within the same circuit and among different brain regions. At the ultrastructural level, they differ in their spatial contact to synaptic components, which may contribute to the circuit‐specific properties of synaptic transmission (Figure 1). Neuronal cues drive astrocyte morphogenesis and may contribute to their diversity. Intrinsic autonomous mechanisms are also emerging. The morphological diversity of neurons, for example, pyramidal neurons *vs* interneurons, or long thin spines *vs* mushroom spines, is well defined and is correlated with their distinct functions in the CNS. However, the definition of astrocyte morphological identity remains obscure. Single‐cell in situ transcriptomic mapping would greatly advance our understanding of the origin, basis, and functional consequences of morphological heterogeneity of astrocytes.

## 3 MORPHOLOGICAL PLASTICITY OF ASTROCYTES

### 3.1 Phenotypes

Astrocytes show structural plasticity in response to synaptic activity and behavior, which in turn contributes to the remodeling of the surrounding synapses. Therefore, understanding astrocyte structural plasticity is essential toward to deciphering the molecular basis for learning, memory, and other brain activities.

Astrocytes show rapid, within few hours, and reversible structural remodeling that occurs in PAPs to change the extent of the coverage of neutrophil in response to strong behavioral stimuli, like parturition, lactation, osmotic stimulation, and stress.^49^ Astrocyte structural remodeling also occurs during the switch of brain states. During natural sleep and general anesthesia, increased ECS volume was observed while opposite changes in ECS volume were detected in arousal and recovery of general anesthesia.^17^, ^50^ These state‐dependent changes in ECS volume are modulated by PAPs plasticity (reviewed in ref 51). Thus, astrocytes may be involved in the on‐off switch for consciousness, a process that has been enigmatic. In the mediobasal hypothalamus, the high‐order processes of astrocytes displayed shortening in fasting and elongation in fed status.^52^ Astrocyte structural remodeling is also instructed by neuronal activities evidenced by both in vitro slice preparation^53^, ^54^, ^55^ and in vivo detection.^16^, ^18^ For instance, synaptic activation that induces hippocampal LTP or in vivo whisker stimulation is sufficient to induce a rapid, within dozens of minutes, PAPs motility, accompanied with increased astrocytic coverage of spines.^16^, ^18^ In short, PAPs are highly dynamic structures, of which the plasticity can occur from minutes to hours under the brain activities in various regions.

### 3.2 Molecular basis

Recent studies have revealed that PAPs plasticity can be regulated by synaptic activity. Early electron microscopy data revealed that sustained whisker stimulation elicited significant increase in the astrocytic ensheathment of excitatory synapses on dendritic spines with increased glutamate transporter expression.^56^ This finding is further confirmed by in vivo observations in mouse somatosensory cortex.^16^, ^18^ Interestingly, the activity‐related structural plasticity of PAPs seems to be glutamate‐ and action potential‐dependent, as well as metabotropic glutamate receptors (mGluRs) mediated, but independent from GABAergic transmission.^57^ However, the basal motility of PAPs may be independent of action potentials.^58^ Structure plasticity of primary astrocytes was simulated by application of mGluR 3 and 5 agonists, but abolished by a combination of glutamate with mGluR antagonists.^19^ These findings were further confirmed by investigation performed in the hippocampal slices.^16^ Together, these data indicate that neuronal activity‐dependent PAPs plasticity is mediated by mGluRs.

Neuronal activity and mGluR activation induce Ca^2+^ transients in PAPs,^59^, ^60^ and photolysis of caged Ca^2+^ in cultured astrocytes directly triggered a pronounced outgrowth of PAPs,^61^ suggesting the involvement of intracellular Ca^2+^ signals in PAP plasticity. In acute slices from transgenic mice that have an attenuated inositol 1,4,5‐trisphosphate (IP3)‐induced Ca^2+^ release, application of mGluR agonist resulted in reduced Ca^2+^ signals in hippocampal astrocytic processes and decreased Ca^2+^ activity in these mice resulted in reduced PAPs coverage of synapses with elevated proportion of uncovered synapses.^62^ Similarly, diminishing of astrocyte intracellular Ca^2+^ transients with BAPTA significantly suppressed the PAPs motility. In contrast, increased PAPs motility was detected by selectively induced Ca^2+^ transients in astrocytes using the astrocyte‐specific expression of exogenous G_q_‐coupled receptors.^16^

Actin remodeling is a primary driving force for morphological plasticity of peripheral extremities in a variety of cell types.^53^, ^57^ The membrane cytoskeleton linker Ezrin is enriched in astrocytes,^63^, ^64^ especially located in PAPs, but not GFAP‐labeled main branches.^19^, ^65^ Interestingly, filopodia formation and motility require Ezrin in the membrane/cytoskeleton bound form which is glutamate‐induced and Ca^2+^‐dependent,^66^ as revealed by applying Ezrin siRNA or dominant‐negative Ezrin in primary astrocytes.^19^ Ca^2+^‐dependent PAPs plasticity is also mediated by actin‐binding protein Profilin‐1. Overexpression of mutant Profilin‐1 in cultured astrocytes fully suppressed PAPs motility induced by photolysis of caged Ca^2+ ^release.^61^ In addition, the Rho family of GTPases is also known to regulate the actin cytoskeleton through various pathways and thus control cell morphology. Members of the Rho family of GTPases, such as RhoA, Cdc42, Rac1, and the Rho‐associated kinase (ROCK), are expressed in astrocytes and required to the structural plasticity of astrocytes (reviewed in ref.^67^). This evidence indicates that astrocytic Ca^2+^‐dependent actin remodeling mechanisms are key regulators for PAPs plasticity.

**Figure 2:**
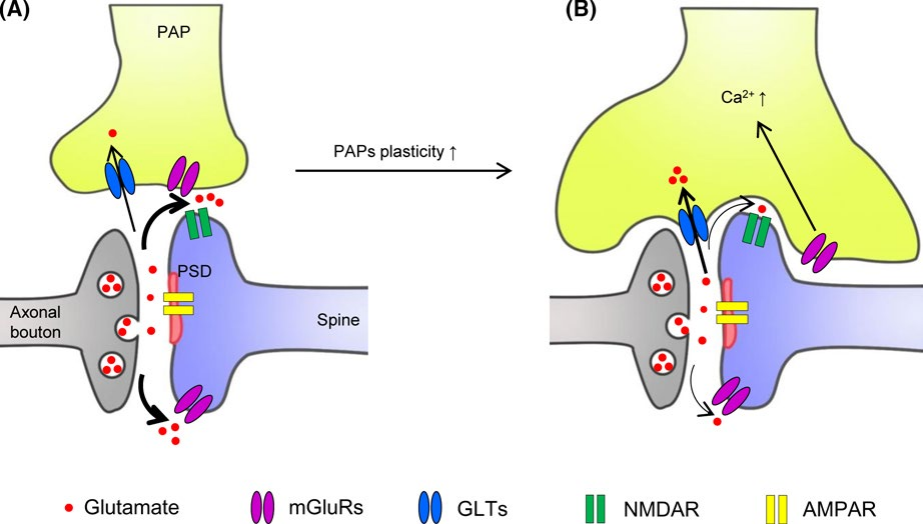
PAPs plasticity regulates the astrocytic coverage of synapse and synaptic transmission. A, A synapse with limited astrocytic coverage with low glutamate uptake by GLTs but high glutamate spillover which facilitates the activation of the extrasynaptic NMDAR and mGluRs. B, Increased PAPs plasticity, induced by mGluRs‐mediated Ca^2+ ^signals, enhances astrocytic coverage of synapse, with increased glutamate uptake by GLTs but decreased glutamate releasing into the extracellular space (ECS) and extrasynaptic activation. AMPAR, α‐amino‐3‐hydroxy‐5‐methyl‐4‐isoxazolepropionic acid receptor; GLTs, glial glutamate transporters, including GLT1 and GLAST; mGluRs, metabotropic glutamate receptors; NMDAR, N‐Methyl‐D‐aspartic acid receptor; PSD, postsynaptic density

Alternative pathways/mechanisms are reported to participate in metabolic‐ and state‐dependent astrocytic structure plasticity. Structure plasticity of astrocytes during metabolic physiology is mediated by IKKb/NF‐kB pathway. Chronic overnutrition induced astrocytic plasticity impairment, with sustained shortening of high‐order processes, was simulated by upregulation of astrocytic IKKb/NF‐kB but prevented by appropriate inhibition in astrocytic IKKb/NF‐kB.^52^ By contrast, state‐dependent PAPs plasticity is likely mediated by swelling of astrocytic processes (reviewed in ref.^51^, ^68^). Neuronal excitation during wakefulness and general anesthesia recovery is accompanied by increased extracellular K^+^ concentration,^17^ and astrocytic K^+^ uptake with the correlated H_2_O influx causes cell swelling.^51^, ^68^ Importantly, involvement of β‐adrenergic receptors (βARs) is also reported. Concerted release of norepinephrine mediates arousal^69^; and activation of βARs, through increased cytosolic cAMP concentration, expand the astrocytic wrapping of the neuropil and reduce ECS volume.^70^ Therefore, astrocyte PAPs plasticity is diverse in phenotypes, and multiple mechanisms/pathways may underlie different phenotypes depending on physiological conditions (Figure 2).

### 3.3 Functional implications

It is well recognized that PAPs plasticity modulates the synaptic efficacy,^33^, ^71^ spine stability,^53^ and synaptic maturation.^16^, ^18^ However, how PAPs plasticity contributes to behaviors? Direct manipulation of astrocyte structural plasticity in a specialized circuit with well‐defined behavior output is needed to answer this question. So far, specific genetic tools controlling astrocyte structural plasticity are scarcely available. Targeting key molecular machineries such as Ezrin and Rho family of GTPases seems to be promising strategies to start. Noninvasive methods such as optogenetics/chemogenetics designed for specific manipulation of astrocyte PAPs mobility would facilitate one to dissect the roles of PAPs plasticity in behaviors, such as circadian rhythm,^72^ learning/memory,^25^, ^73^, ^74^ wakefulness/sleep,^75^, ^76^ and general anesthesia,^17^, ^77^ in which astrocytes actively participate.

## 4 ASTROCYTE MORPHOLOGICAL DEFICITS IN NEURODEGENERATIVE DISEASES

Astrocytes become reactive in response to injury or other pathological processes. Upregulation of GFAP, the main constituent of astrocyte intermediate filaments, has been considered as a hallmark of reactive astrocytes in primates and rodents.^20^, ^21^, ^22^ The increased number and length of GFAP‐positive processes revealed by GFAP immunostaining has been found in a variety of pathological conditions and has been often used to define astrocytic morphological changes such as cellular hypertrophy.^78^ However, GFAP staining only reveals the cytoskeletal structure but not the entire cellular morphology, thus cytoskeletal hypertrophy would be a better term to describe astrocyte reactivity while GFAP expression is concerned. Indeed, detailed morphological analysis of astrocytes in neurotrauma models revealed a remarkable increase in GFAP expression with little change in the extent of overlap between the unique domains of individual astrocytes, and no apparent cellular hypertrophy was found.^79^

### 4.1 Alzheimer's disease (AD)

Although cytoskeletal hypertrophy and upregulation of GFAP were found in the late stages of AD mouse models and in human postmortem tissue,^80^ at the onset stages of AD in the 3xTg‐AD mice and in the PDAPP‐J20 mice, there is a progressive astrocytic atrophy with decreased GFAP staining in the cortex and hippocampus.^81^, ^82^ The switch from atrophic phenotype at the early stage to later hypertrophic phenotype may be associated with the formation of amyloid plaques, since only the astrocytes directly associated with amyloid plaques became hypertrophic. It is well accepted that astrogliosis in the late and terminal stages contributes to beta‐amyloid uptake and degradation.^83^, ^84^ By contrast, little is known regarding the molecular basis for astrocyte atrophy preceding astrogliosis, and how it contributes to the onset of AD. It is noteworthy that vascular defects at the early stages of AD have been detected including reduced blood flow and altered morphology in the brains of AD patients^85^, ^86^, ^87^ and astrocytes play integral roles through their PAPs in neurovascular units by sensing neuronal activities and controlling vasoconstriction and vasodilatation.^88^ It is thus possible that the astrocyte atrophy may contribute to the damage of neurovascular unit at the onset of AD and thereby contribute to the progression of the disease.

### 4.2 Parkinson's disease (PD)

Emerging evidence suggests that astrocytes are involved in PD. Several PD‐related genes including *PARK7*, *PARK2,* and *SNCA* were found to play important roles in astrocyte biology and contribute to PD pathophysiology.^89^ However, astrocyte morphological changes in the progression of PD remain obscure. Studies on autopsies of the substantia nigra and putamen from Parkinson's disease patients revealed little or only mild reactive astrogliosis by using GFAP staining.^90^ Interestingly, at the ultrastructural level, in the striatum of MPTP‐treated parkinsonian monkeys, there is a significant expansion of the coverage by astrocyte processes of striatal vGluT1‐ or vGluT2‐positive glutamatergic synapses in the parkinsonian state.^91^ From these studies, it seems that the extent of astrocyte ensheathment of pre‐ and postsynaptic components was increased in PD with a larger astrocyte process surface area and volume surrounding synapses. The underlying molecular basis and the significance of the astrocyte structural changes are unclear but may have important implications for the altered synaptic strength, plasticity, and excitotoxicity in PD.

### 4.3 Huntington's Disease (HD)

**Figure 3:**
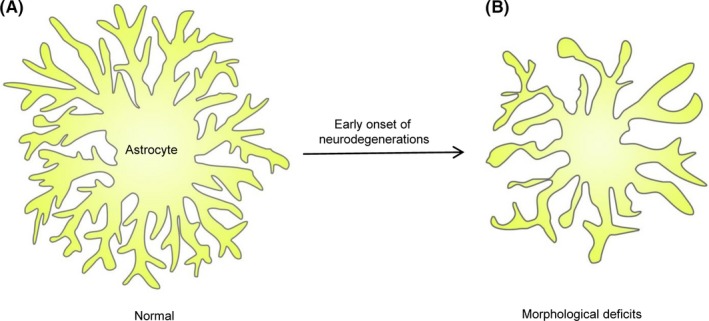
Morphological deficits of astrocytes in the early onset of neurodegeneration. A, Cartoon presentation of an astrocyte in normal physiology condition. B, An astrocyte in the early onset of neurodegenerations with decreased fine processes and territory volume

Accumulation of mutant huntingtin protein (mHTT) in cortical and striatal astrocytes was detected in brains from HD patients and in mouse models of HD, which disrupts astrocyte glutamate transporter expression and contributes to HD pathology.^92^ In the early stage of HD, several astrocyte dysfunctions have been revealed: (a) reduced K^+^ buffering mediated by the downregulation of astrocyte Kir4.1 in two mouse models for HD, which contributes to increased extracellular K^+^ in vivo and the enhanced neuronal excitability in striatal medium‐sized spiny neurons (MSN)^93^; (b) disrupted astrocyte intracellular Ca^2+^ signaling and extracellular glutamate uptake mediated by GLT1, which could be rescued partially by Kir4.1 overexpression in R6/2 mice.^94^ Importantly, by limiting the lateral charge transfer or by providing the driving force, Kir4.1 conductance modulates the activity of GLT1.^95^ It is also noteworthy that Kir4.1 loss of functions have been found in a variety of neurological disorders including AD,^96^ amyotrophic lateral sclerosis (ALS),^97^, ^98^ epilepsy,^99^ and depression.^100^ In parallel, astrocytes did not show apparent change in GFAP expression,^23^, ^92^ suggesting no apparent astrogliosis. However, they did exhibit profound morphological deficits including reduced PAP volumes, territory size, and altered proximity of astrocyte processes to cortical and thalamic excitatory inputs revealed by the astrocyte‐synapse proximity reporter NAPA.^23^ Therefore, both homeostatic dysfunctions and morphological deficits were identified in neurodegeneration including HD (Figure 3). Further studies are needed to test if the correction of the homeostatic dysfunction would rescue astrocyte morphology and *vice versa*.

Although to be experimentally verified, it is likely that the deficits in the physical contacts between astrocyte and synapses contribute to the homeostatic dysfunctions of astrocytes in neurodegenerations. For instance, neuronal excitotoxicity represents one of the common mechanisms that involve astrocyte homeostatic dysfunction in AD, PD, and HD. Restoration of astrocyte‐synapse contact would enhance glutamate and K^+ ^uptake and reduce spillover to reduce excitotoxicity. Thus, astrocyte morphological integrity may represent an important therapeutic target. Specifically, what are the general mechanisms, if any, underlying astrocyte morphological deficits at the early stage of neurodegeneration? Do the astrocyte morphological deficits trigger astrocyte functional deficits, particularly in ion and transmitter homeostasis? Can one rescue or slow down disease phenotypes by rescuing astrocyte morphology? The answers to questions above would be paramount to further our understanding of astroglial involvement in neurological diseases and may open new avenues to the clinical treatment of brain diseases.

## CONFLICT OF INTEREST

The authors declare no conflicts of interest.

## References

[1] Herculano‐Houzel S . The glia/neuron ratio: how it varies uniformly across brain structures and species and what that means for brain physiology and evolution. Glia. 2014;62(9):1377‐1391. 24807023 10.1002/glia.22683

[2] Gallo V , Deneen B . Glial development: the crossroads of regeneration and repair in the CNS. Neuron. 2014;83(2):283‐308. 25033178 10.1016/j.neuron.2014.06.010 PMC4114724

[3] Colombo JA , Reisin HD . Interlaminar astroglia of the cerebral cortex: a marker of the primate brain. Brain Res. 2004;1006(1):126‐131. 15047031 10.1016/j.brainres.2004.02.003

[4] Rodnight RB , Gottfried C . Morphological plasticity of rodent astroglia. J Neurochem. 2013;124(3):263‐275. 23278277 10.1111/jnc.12087

[5] Lundgaard I , Osório Mj , Kress Bt , Sanggaard S , Nedergaard M . White matter astrocytes in health and disease. Neuroscience. 2014;276:161‐173. 24231735 10.1016/j.neuroscience.2013.10.050 PMC4016995

[6] Emsley JG , Macklis JD . Astroglial heterogeneity closely reflects the neuronal‐defined anatomy of the adult murine CNS. Neuron Glia Biol. 2006;2(3):175‐186. 17356684 10.1017/S1740925X06000202 PMC1820889

[7] Farhy‐Tselnicker I , Allen NJ . Astrocytes, neurons, synapses: a tripartite view on cortical circuit development. Neural Dev. 2018;13(1):7. 29712572 10.1186/s13064-018-0104-y PMC5928581

[8] Bushong EA , Martone ME , Jones YZ , Ellisman MH . Protoplasmic astrocytes in CA1 stratum radiatum occupy separate anatomical domains. J Neurosci. 2002;22(1):183‐192. 11756501 10.1523/JNEUROSCI.22-01-00183.2002 PMC6757596

[9] Ogata K , Kosaka T . Structural and quantitative analysis of astrocytes in the mouse hippocampus. Neuroscience. 2002;113(1):221‐233. 12123700 10.1016/s0306-4522(02)00041-6

[10] Chai H , Diaz‐Castro B , Shigetomi E , et al. Neural Circuit‐Specialized Astrocytes: Transcriptomic, Proteomic, Morphological, and Functional Evidence. Neuron. 2017;95(3):531‐549. e539. 28712653 10.1016/j.neuron.2017.06.029 PMC5811312

[11] Khakh BS , Sofroniew MV . Diversity of astrocyte functions and phenotypes in neural circuits. Nat Neurosci. 2015;18(7):942‐952. 26108722 10.1038/nn.4043 PMC5258184

[12] Oberheim Na , Takano T , Han X , et al. Uniquely hominid features of adult human astrocytes. J Neurosci. 2009;29(10):3276‐3287. 19279265 10.1523/JNEUROSCI.4707-08.2009 PMC2819812

[13] Oberheim NA , Wang X , Goldman S , Nedergaard M . Astrocytic complexity distinguishes the human brain. Trends Neurosci. 2006;29(10):547‐553. 16938356 10.1016/j.tins.2006.08.004

[14] Han X , Chen M , Wang F , et al. Forebrain engraftment by human glial progenitor cells enhances synaptic plasticity and learning in adult mice. Cell Stem Cell. 2013;12(3):342‐353. 23472873 10.1016/j.stem.2012.12.015 PMC3700554

[15] Lanjakornsiripan D , Pior B‐J , Kawaguchi D , et al. Layer‐specific morphological and molecular differences in neocortical astrocytes and their dependence on neuronal layers. Nat Commun. 2018;9(1):1623. 29691400 10.1038/s41467-018-03940-3 PMC5915416

[16] Bernardinelli Y , Randall J , Janett E , et al. Activity‐dependent structural plasticity of perisynaptic astrocytic domains promotes excitatory synapse stability. Curr Biol. 2014;24(15):1679‐1688. 25042585 10.1016/j.cub.2014.06.025

[17] Ding F , O’Donnell J , Qiwu Xu NK , et al. Changes in the composition of brain interstitial ions control the sleep‐wake cycle. Science. 2016;352(6285):550‐555. 27126038 10.1126/science.aad4821 PMC5441687

[18] Perez‐Alvarez A , Navarrete M , Covelo A , Martin ED , Araque A . Structural and functional plasticity of astrocyte processes and dendritic spine interactions. J Neurosci. 2014;34(38):12738‐12744. 25232111 10.1523/JNEUROSCI.2401-14.2014 PMC6705321

[19] Lavialle M , Aumann G , Anlauf E , Prols F , Arpin M , Derouiche A . Structural plasticity of perisynaptic astrocyte processes involves ezrin and metabotropic glutamate receptors. Proc Natl Acad Sci USA. 2011;108(31):12915‐12919. 21753079 10.1073/pnas.1100957108 PMC3150955

[20] Ceyzériat K , Ben Haim L , Denizot A , et al. Modulation of astrocyte reactivity improves functional deficits in mouse models of Alzheimer's disease. Acta Neuropathol Commun. 2018;6(1):104. 30322407 10.1186/s40478-018-0606-1 PMC6190663

[21] Molofsky Av , Krenick R , Ullian E , et al. Astrocytes and disease: a neurodevelopmental perspective. Genes Dev. 2012;26(9):891‐907. 22549954 10.1101/gad.188326.112 PMC3347787

[22] Burda JE , Sofroniew MV . Reactive gliosis and the multicellular response to CNS damage and disease. Neuron. 2014;81(2):229‐248. 24462092 10.1016/j.neuron.2013.12.034 PMC3984950

[23] Octeau JC , Chai H , Jiang R , et al. An Optical Neuron‐Astrocyte Proximity Assay at Synaptic Distance Scales. Neuron. 2018;98(1):49‐66. e49. 29621490 10.1016/j.neuron.2018.03.003 PMC5916847

[24] Halassa MM , Fellin T , Takano H , Dong J‐H , Haydon PG . Synaptic islands defined by the territory of a single astrocyte. J Neurosci. 2007;27(24):6473‐6477. 17567808 10.1523/JNEUROSCI.1419-07.2007 PMC6672436

[25] Diniz DG , de Oliveira MA , de Lima CM , et al. Age, environment, object recognition and morphological diversity of GFAP‐immunolabeled astrocytes. Behav Brain Funct. 2016;12(1):28. 27719674 10.1186/s12993-016-0111-2 PMC5056502

[26] Bernardinelli Y , Muller D , Nikonenko I . Astrocyte‐synapse structural plasticity. Neural Plast. 2014;2014:232105. 24511394 10.1155/2014/232105 PMC3910461

[27] Witcher MR , Kirov SA , Harris KM . Plasticity of perisynaptic astroglia during synaptogenesis in the mature rat hippocampus. Glia. 2007;55(1):13‐23. 17001633 10.1002/glia.20415

[28] Ventura R , Harris KM . Three‐dimensional relationships between hippocampal synapses and astrocytes. J Neurosci. 1999;19(16):6897‐6906. 10436047 10.1523/JNEUROSCI.19-16-06897.1999 PMC6782870

[29] Gavrilov N , Golyagina I , Brazhe A , Scimemi A , Turlapov V , Semyanov A . Astrocytic Coverage of Dendritic Spines, Dendritic Shafts, and Axonal Boutons in Hippocampal Neuropil. Front Cell Neurosci. 2018;12:248. 30174590 10.3389/fncel.2018.00248 PMC6108058

[30] Rollenhagen A , Satzler K , Rodriguez Ep , Jonas P , Frotscher M , Lubke J . Structural determinants of transmission at large hippocampal mossy fiber synapses. J Neurosci. 2007;27(39):10434‐10444. 17898215 10.1523/JNEUROSCI.1946-07.2007 PMC6673150

[31] Xu‐Friedman MA , Harris KM , Regehr WG . Three‐dimensional comparison of ultrastructural characteristics at depressing and facilitating synapses onto cerebellar Purkinje cells. J Neurosci. 2001;21(17):6666‐6672. 11517256 10.1523/JNEUROSCI.21-17-06666.2001 PMC6763067

[32] Piet R , Vargová L , Syková E , Poulain DA , Oliet S . Physiological contribution of the astrocytic environment of neurons to intersynaptic crosstalk. Proc Natl Acad Sci USA. 2004;101(7):2151‐2155. 14766975 10.1073/pnas.0308408100 PMC357067

[33] Oliet S , Piet R , Poulain DA , Theodosis DT . Glial modulation of synaptic transmission: Insights from the supraoptic nucleus of the hypothalamus. Glia. 2004;47(3):258‐267. 15252815 10.1002/glia.20032

[34] Dvorzhak A , Melnick I , Grantyn R . Astrocytes and presynaptic plasticity in the striatum: Evidence and unanswered questions. Brain Res Bull. 2018;136:17‐25. 28069435 10.1016/j.brainresbull.2017.01.001

[35] Lushnikova I , Skibo G , Muller D , Nikonenko I . Synaptic potentiation induces increased glial coverage of excitatory synapses in CA1 hippocampus. Hippocampus. 2009;19(8):753‐762. 19156853 10.1002/hipo.20551

[36] Medvedev N , Popov V , Henneberger C , Kraev I , Rusakov Da , Stewart Mg . Glia selectively approach synapses on thin dendritic spines. Philos Trans R Soc Lond B Biol Sci. 2014;369(1654):20140047. 25225105 10.1098/rstb.2014.0047 PMC4173297

[37] Pannasch U , Freche D , Dallérac G , et al. Connexin 30 sets synaptic strength by controlling astroglial synapse invasion. Nat Neurosci. 2014;17(4):549‐558. 24584052 10.1038/nn.3662

[38] Stogsdill JA , Ramirez J , Liu Di , et al. Astrocytic neuroligins control astrocyte morphogenesis and synaptogenesis. Nature. 2017;551(7679):192‐197. 29120426 10.1038/nature24638 PMC5796651

[39] Ichtchenko K , Nguyen T , Südhof TC . Structures, alternative splicing, and neurexin binding of multiple neuroligins. J Biol Chem. 1996;271(5):2676‐2682. 8576240 10.1074/jbc.271.5.2676

[40] Reissner C , Runkel F , Missler M . Neurexins. Genome Biol. 2013;(14):213. 24083347 10.1186/gb-2013-14-9-213 PMC4056431

[41] Nagy Ji , Patel D , Ochalski P , Stelmack Gl . Connexin30 in rodent, cat and human brain: selective expression in gray matter astrocytes, co‐localization with connexin43 at gap junctions and late developmental appearance. Neuroscience. 1999;88(2):447‐468. 10197766 10.1016/s0306-4522(98)00191-2

[42] Ghezali G , Calvo CF , Pillet LE , et al. Connexin 30 controls astroglial polarization during postnatal brain development. Development. 2018;145(4). 10.1242/dev.155275 PMC5869003 29475972

[43] Houades V , Koulakoff A , Ezan P , Seif I , Giaume C . Gap junction‐mediated astrocytic networks in the mouse barrel cortex. J Neurosci. 2008;28(20):5207‐5217. 18480277 10.1523/JNEUROSCI.5100-07.2008 PMC6670639

[44] Agarwal A , Bergles DE . Astrocyte morphology is controlled by neuron‐derived FGF. Neuron. 2014;83(2):255‐257. 25033173 10.1016/j.neuron.2014.07.005 PMC4102929

[45] Stork T , Sheehan A , Tasdemir‐Yilmaz O , Freeman M . Neuron‐glia interactions through the Heartless FGF receptor signaling pathway mediate morphogenesis of Drosophila astrocytes. Neuron. 2014;83(2):388‐403. 25033182 10.1016/j.neuron.2014.06.026 PMC4124900

[46] Wu B , Li J , Chou YH , et al. Fibroblast growth factor signaling instructs ensheathing glia wrapping of Drosophila olfactory glomeruli. Proc Natl Acad Sci USA. 2017;114(29):7505‐7512. 28674010 10.1073/pnas.1706533114 PMC5530699

[47] Richier B , Vijandi CdM , Mackensen S , Salecker I . Lapsyn controls branch extension and positioning of astrocyte‐like glia in the Drosophila optic lobe. Nat Commun. 2017;8(1):317. 28827667 10.1038/s41467-017-00384-z PMC5567088

[48] Cho S , Muthukumar AK , Stork T , Coutinho‐Budd JC , Freeman MR . Focal adhesion molecules regulate astrocyte morphology and glutamate transporters to suppress seizure‐like behavior. Proc Natl Acad Sci USA. 2018;115(44):11316‐11321. 30327343 10.1073/pnas.1800830115 PMC6217442

[49] Theodosis DT . Oxytocin‐secreting neurons: A physiological model of morphological neuronal and glial plasticity in the adult hypothalamus. Front Neuroendocrinol. 2002;23(1):101‐135. 11906204 10.1006/frne.2001.0226

[50] Xie L , Kang H , Xu Q , et al. Sleep drives metabolite clearance from the adult brain. Science. 2013;342(6156):373‐377. 24136970 10.1126/science.1241224 PMC3880190

[51] Sykova E , Nicholson C . Diffusion in brain extracellular space. Physiol Rev. 2008;88(4):1277‐1340. 18923183 10.1152/physrev.00027.2007 PMC2785730

[52] Zhang Y , Reichel JM , Han C , et al. Astrocytic Process Plasticity and IKKbeta/NF‐kappaB in Central Control of Blood Glucose, Blood Pressure, and Body Weight. Cell Metab. 2017;25(5):1091‐1102. e1094. 28467927 10.1016/j.cmet.2017.04.002 PMC5576872

[53] Nishida H , Okabe S . Direct astrocytic contacts regulate local maturation of dendritic spines. J Neurosci. 2007;27(2):331‐340. 17215394 10.1523/JNEUROSCI.4466-06.2007 PMC6672072

[54] Zhou L , Martinez SJ , Haber M , et al. EphA4 signaling regulates phospholipase Cgamma1 activation, cofilin membrane association, and dendritic spine morphology. J Neurosci. 2007;27(19):5127‐5138. 17494698 10.1523/JNEUROSCI.1170-07.2007 PMC6672384

[55] Hirrlinger J , Hulsmann S , Kirchhoff F . Astroglial processes show spontaneous motility at active synaptic terminals in situ. Eur J Neurosci. 2004;20(8):2235‐2239. 15450103 10.1111/j.1460-9568.2004.03689.x

[56] Genoud C , Quairiaux C , Steiner P , et al. Plasticity of astrocytic coverage and glutamate transporter expression in adult mouse cortex. PLoS Biol. 2006;4(11):e343. 17048987 10.1371/journal.pbio.0040343 PMC1609127

[57] Haber M , Zhou L , Murai KK . Cooperative astrocyte and dendritic spine dynamics at hippocampal excitatory synapses. J Neurosci. 2006;26(35):8881‐8891. 16943543 10.1523/JNEUROSCI.1302-06.2006 PMC6675342

[58] Verbich D , Prenosil GA , Chang P‐Y , Murai KK , McKinney RA . Glial glutamate transport modulates dendritic spine head protrusions in the hippocampus. Glia. 2012;60(7):1067‐1077. 22488940 10.1002/glia.22335

[59] Panatier A , Vallée J , Haber M , Murai K , Lacaille J‐C , Robitaille R . Astrocytes are endogenous regulators of basal transmission at central synapses. Cell. 2011;146(5):785‐798. 21855979 10.1016/j.cell.2011.07.022

[60] Shigetomi E , Patel S , Khakh BS . Probing the Complexities of Astrocyte Calcium Signaling. Trends Cell Biol. 2016;26(4):300‐312. 26896246 10.1016/j.tcb.2016.01.003 PMC4946798

[61] Molotkov D , Zobova S , Arcas JM , et al. Calcium‐induced outgrowth of astrocytic peripheral processes requires actin binding by Profilin‐1. Cell Calcium. 2013;53(5–6):338‐348. 23578580 10.1016/j.ceca.2013.03.001

[62] Tanaka M , Shih PY , Gomi H . et al. Astrocytic Ca2+ signals are required for the functional integrity of tripartite synapses. Mol Brain. 2013;(6):6. 23356992 10.1186/1756-6606-6-6 PMC3563617

[63] Grönholm M , Teesalu T , Tyynelä J , et al. Characterization of the NF2 protein merlin and the ERM protein ezrin in human, rat, and mouse central nervous system. Mol Cell Neurosci. 2005;28(4):683‐693. 15797715 10.1016/j.mcn.2004.11.014

[64] Cahoy Jd , Emery B , Kaushal A , et al. A transcriptome database for astrocytes, neurons, and oligodendrocytes: a new resource for understanding brain development and function. J Neurosci. 2008;28(1):264‐278. 18171944 10.1523/JNEUROSCI.4178-07.2008 PMC6671143

[65] Derouiche A , Frotscher M . Peripheral astrocyte processes: monitoring by selective immunostaining for the actin‐binding ERM proteins. Glia. 2001;36(3):330‐341. 11746770 10.1002/glia.1120

[66] Hs K , Cd B , J P. . Glutamate receptor‐mediated phosphorylation of ezrin/radixin/moesin proteins is implicated in filopodial protrusion of primary cultured hippocampal neuronal cells. J Neurochem. 2010;113(6):1565‐1576. 20367752 10.1111/j.1471-4159.2010.06713.x

[67] Zeug A , Müller FE , Anders S , et al. Control of astrocyte morphology by Rho GTPases. Brain Res Bull. 2018;136:44‐53. 28502648 10.1016/j.brainresbull.2017.05.003

[68] Nicholson C , Hrabetova S . Brain Extracellular Space: The Final Frontier of Neuroscience. Biophys J. 2017;113(10):2133‐2142. 28755756 10.1016/j.bpj.2017.06.052 PMC5700249

[69] Saper CB , Scammell TE , Lu J . Hypothalamic regulation of sleep and circadian rhythms. Nature. 2005;437(7063):1257‐1263. 16251950 10.1038/nature04284

[70] Sherpa AD , Xiao F , Joseph N , et al. Activation of beta‐adrenergic receptors in rat visual cortex expands astrocytic processes and reduces extracellular space volume. Synapse. 2016;70(8):307‐316. 27085090 10.1002/syn.21908 PMC4909535

[71] Sh O , R P, DA P. . Control of glutamate clearance and synaptic efficacy by glial coverage of neurons. Science. 2001;292(5518):923‐926. 11340204 10.1126/science.1059162

[72] Brancaccio M , Edwards MD , Patton AP , et al. Cell‐autonomous clock of astrocytes drives circadian behavior in mammals. Science. 2019;363(6423):187‐192. 30630934 10.1126/science.aat4104 PMC6440650

[73] E D, MA DS‐S, C F, , et al. Connexin30‐deficient mice show increased emotionality and decreased rearing activity in the open‐field along with neurochemical changes. Eur. J. Neurosci. 2003;18(3):629‐638. 12911759 10.1046/j.1460-9568.2003.02784.x

[74] Alberini CM , Cruz E , Descalzi G , et al. Astrocyte glycogen and lactate: New insights into learning and memory mechanisms. Glia. 2018;66(6):1244‐1262. 29076603 10.1002/glia.23250 PMC5903986

[75] Papouin T , Dunphy JM , Tolman M , et al. Septal Cholinergic Neuromodulation Tunes the Astrocyte‐Dependent Gating of Hippocampal NMDA Receptors to Wakefulness. Neuron. 2017;94(4):pp. 840‐854 e847. 10.1016/j.neuron.2017.04.021 PMC5484087 28479102

[76] Poskanzer KE , Yuste R . Astrocytes regulate cortical state switching in vivo. Proceedings of the National Academy of Sciences. 2016;113(19):E2675‐E2684. 10.1073/pnas.1520759113 PMC4868485 27122314

[77] Thrane As , Rangroo Thrane V , Zeppenfeld D , et al. General anesthesia selectively disrupts astrocyte calcium signaling in the awake mouse cortex. Proc Natl Acad Sci USA. 2012;109(46):18974–18979. 23112168 10.1073/pnas.1209448109 PMC3503159

[78] Pekny M , Pekna M . Astrocyte reactivity and reactive astrogliosis: costs and benefits. Physiol Rev. 2014;94(4):1077‐1098. 25287860 10.1152/physrev.00041.2013

[79] Wilhelmsson U , Bushong Ea , Price Dl , et al. Redefining the concept of reactive astrocytes as cells that remain within their unique domains upon reaction to injury. Proc Natl Acad Sci USA. 2006;103(46):17513‐17518. 17090684 10.1073/pnas.0602841103 PMC1859960

[80] Simpson Je , Ince Pg , Lace G , et al. Astrocyte phenotype in relation to Alzheimer‐type pathology in the ageing brain. Neurobiol Aging. 2010;31(4):578‐590. 18586353 10.1016/j.neurobiolaging.2008.05.015

[81] Beauquis J , Pavía P , Pomilio C , et al. Environmental enrichment prevents astroglial pathological changes in the hippocampus of APP transgenic mice, model of Alzheimer's disease. Exp Neurol. 2013;239:28‐37. 23022919 10.1016/j.expneurol.2012.09.009

[82] Yeh C‐Y , Vadhwana B , Verkhratsky A , Rodríguez JJ . Early astrocytic atrophy in the entorhinal cortex of a triple transgenic animal model of Alzheimer's disease. ASN Neuro. 2011;3(5):271‐279. 22103264 10.1042/AN20110025 PMC3243908

[83] Guénette SY . Astrocytes: a cellular player in Aβ clearance and degradation. Trends Mol Med. 2003;9(7):279‐280. 12900212 10.1016/s1471-4914(03)00112-6

[84] Nicoll JA , Weller RO . A new role for astrocytes: beta‐amyloid homeostasis and degradation. Trends Mol Med. 2003;9(7):281‐282. 12900213 10.1016/s1471-4914(03)00109-6

[85] Iadecola C . Neurovascular regulation in the normal brain and in Alzheimer's disease. Nat Rev Neurosci. 2004;5(5):347‐360. 15100718 10.1038/nrn1387

[86] Bell RD , Zlokovic BV . Neurovascular mechanisms and blood‐brain barrier disorder in Alzheimer's disease. Acta Neuropathol. 2009;118(1):103‐113. 19319544 10.1007/s00401-009-0522-3 PMC2853006

[87] Farkas E , Luiten PG . Cerebral microvascular pathology in aging and Alzheimer's disease. Prog Neurobiol. 2001;64(6):575‐611. 11311463 10.1016/s0301-0082(00)00068-x

[88] Iadecola C , Nedergaard M . Glial regulation of the cerebral microvasculature. Nat Neurosci. 2007;10(11):1369‐1376. 17965657 10.1038/nn2003

[89] Booth H , Hirst WD , Wade‐Martins R . The Role of Astrocyte Dysfunction in Parkinson's Disease Pathogenesis. Trends Neurosci. 2017;40(6):358‐370. 28527591 10.1016/j.tins.2017.04.001 PMC5462417

[90] Tong J , Ang LC , Williams B , et al. Low levels of astroglial markers in Parkinson's disease: relationship to alpha‐synuclein accumulation. Neurobiol Dis. 2015;82:243‐253. 26102022 10.1016/j.nbd.2015.06.010 PMC4641013

[91] Villalba RM , Smith Y . Neuroglial plasticity at striatal glutamatergic synapses in Parkinson's disease. Front Syst Neurosci. 2011;5:68. 21897810 10.3389/fnsys.2011.00068 PMC3159891

[92] Khakh BS , Beaumont V , Cachope R , Munoz‐Sanjuan I , Goldman SA , Grantyn R . Unravelling and Exploiting Astrocyte Dysfunction in Huntington's Disease. Trends Neurosci. 2017;40(7):422‐437. 28578789 10.1016/j.tins.2017.05.002 PMC5706770

[93] Tong X , Ao Y , Faas GC , et al. Kir4.1 ion channel deficits contribute to neuronal dysfunction in Huntington's disease model mice. Nat Neurosci. 2014;17(5):694‐703. 24686787 10.1038/nn.3691 PMC4064471

[94] Jiang R , Diaz‐Castro B , Looger Ll , Khakh BS . Dysfunctional Calcium and Glutamate Signaling in Striatal Astrocytes from Huntington's Disease Model Mice. J Neurosci. 2016;36(12):3453‐3470. 27013675 10.1523/JNEUROSCI.3693-15.2016 PMC4804005

[95] Dvorzhak A , Vagner T , Kirmse K , et al. Indicators of glutamate transport in single striatal astrocytes and the influence of Kir4.1 in normal and huntington mice. J Neurosci. 2016;36(18):4959‐4975. 27147650 10.1523/JNEUROSCI.0316-16.2016 PMC6601850

[96] Wilcock DM , Vitek MP , Colton CA . Vascular amyloid alters astrocytic water and potassium channels in mouse models and humans with Alzheimer's disease. Neuroscience. 2009;159(3):1055‐1069. 19356689 10.1016/j.neuroscience.2009.01.023 PMC2699894

[97] Kaiser M , Maletzki I , Hülsmann S , et al. Progressive loss of a glial potassium channel (KCNJ10) in the spinal cord of the SOD1 (G93A) transgenic mouse model of amyotrophic lateral sclerosis. J Neurochem. 2006;99(3):900‐912. 16925593 10.1111/j.1471-4159.2006.04131.x

[98] Kelley KW , Ben Haim L , Schirmer L , et al. Astrocyte‐fast motor neuron interactions are required for peak strength. Neuron. 2018;98(2):306‐319. e307. 29606582 10.1016/j.neuron.2018.03.010 PMC5919779

[99] Scholl Ui , Choi M , Liu T , et al. Seizures, sensorineural deafness, ataxia, mental retardation, and electrolyte imbalance (SeSAME syndrome) caused by mutations in KCNJ10. Proc Natl Acad Sci USA. 2009;106(14):5842‐5847. 19289823 10.1073/pnas.0901749106 PMC2656559

[100] Cui Y , Yang Y , Ni Z , et al. Astroglial Kir4.1 in the lateral habenula drives neuronal bursts in depression. Nature. 2018;554(7692):323‐327. 29446379 10.1038/nature25752

